# Real-time evaluation of macozinone activity against *Mycobacterium tuberculosis* through bacterial nanomotion analysis

**DOI:** 10.1128/aac.01318-24

**Published:** 2024-11-27

**Authors:** Anthony Vocat, Amanda Luraschi-Eggemann, Claudia Antoni, Gino Cathomen, Danuta Cichocka, Gilbert Greub, Olga Riabova, Vadim Makarov, Onya Opota, Alfonso Mendoza, Stewart T. Cole, Alexander Sturm

**Affiliations:** 1 Resistell AG, Muttenz, Switzerland; 2 Institute of Microbiology, Lausanne University Hospital and University of Lausanne 536517 , Lausanne, Switzerland; 3 Innovative Medicines for Tuberculosis (iM4TB), Lausanne, Switzerland; 4 Service of Infectious Diseases, Lausanne University Hospital and University of Lausanne 666607 , Lausanne, Switzerland; 5 Research Centre of Biotechnology RAS, Leninsky Prospect, Moscow, Russia; St. George's, University of London, London, United Kingdom

**Keywords:** antibiotic susceptibility test, DprE1 inhibitors, nanomotion, macozinone, *Mycobacterium tuberculosis*

## Abstract

Novel drugs and improved diagnostics for *Mycobacterium tuberculosis* (MTB) are urgently needed and go hand in hand. We evaluated the *in vitro* activity of two benzothiazinone drug candidates (MCZ, PBTZ169; BTZ043) and their main metabolites against MTB using advanced nanomotion technology. The results demonstrated significant reductions in MTB viability within 7 h, indicating the potential for rapid, precise antibiotic susceptibility testing based on a phenotypic read-out in real time. PBTZ169 and H_2_-PBTZ169 achieved 100% separation between the susceptible H37Rv and a resistant *dprE1* mutant strain NTB1. These findings support nanomotion technology’s potential for faster antibiotic susceptibility testing of novel MTB drug candidates targeting the DprE1 enzyme that could reduce empirical treatment duration and antibiotic resistance selection pressure due to inaccurate treatments.

## INTRODUCTION

*Mycobacterium tuberculosis* (MTB) represents a major public health burden in low and middle-income countries (LMICs). The situation is worsened by the emergence of multidrug-resistant (MDR) and extensively drug-resistant (XDR) MTB strains, which severely limit treatment options (1–3). The COVID-19 pandemic has further exacerbated these vulnerabilities, potentially increasing the global MTB burden in the coming years. The World Health Organization considers the development of new antitubercular drugs of critical importance (4). Consequently, new drugs and innovative diagnostic approaches are urgently needed to improve MTB treatment.

Macozinone (MCZ, PBTZ169), currently undergoing phase 2 clinical trials (NCT03334734), along with its relative (BTZ043) (5, 6), targets decaprenylphosphoryl-β-d-ribose-2′-epimerase (DprE1), disrupting arabinogalactan synthesis, a critical component of the mycobacterial cell wall (7). In the patient, both drugs are converted to de-aromatized forms (H_2_-PBTZ169 and H_2_-BTZ043), whose contribution to overall efficacy is unknown.

Effective treatment of MTB relies significantly on accurate diagnostics, typically involving MIC determination of a panel of antibiotics. Current antibiotic susceptibility tests (ASTs) for MTB can take ≥2 weeks (8), necessitating prolonged empirical treatment. Molecular tests like GeneXpert provide faster results for, for example, isoniazid (INH) and rifampicin (RIF) resistance but do not detect susceptibility, providing limited information for treatment optimization.

**Fig 1:**
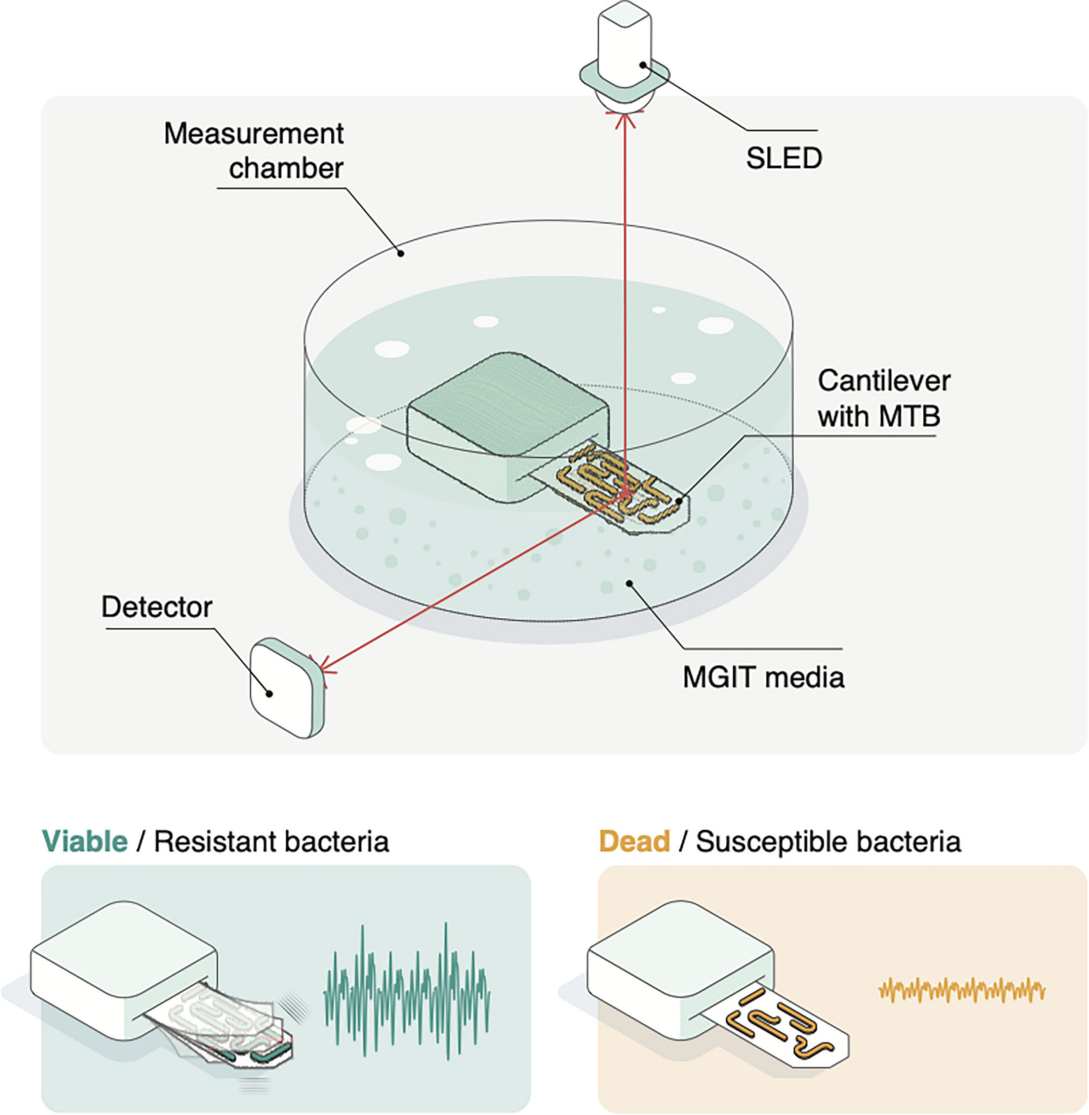
Nanomotion-based antibiotic susceptibility testing (AST) for *M. tuberculosis*. (Top) MGIT-cultured MTB bacilli are attached to a cantilever nanomotion sensor that is submerged in fresh MGIT media. A super-luminescent light emitting diode (SLED) is directed toward the cantilever and reflected onto a position-sensitive photodetector, which monitors its oscillations caused by bacterial nanoscale movements (nanomotion). Viable bacteria cause cantilever oscillations with high amplitudes (bottom left). When exposed to an inhibitory compound, such as the effective anti-TB agent PBTZ169, these bacterial movements diminish and the cantilever oscillations decrease as well (bottom right).

Nanomotion technology, derived from atomic force microscopy (AFM), offers a novel approach to phenotypic ASTs by measuring bacterial viability through cellular vibrations instead of growth (9–14). A nanomechanical sensor, consisting of a functionalized cantilever, oscillates in response to bacterial vibrations related to cellular and metabolic activity (Fig. 1). Drug exposure alters these vibrations, changing the cantilever oscillations, which are detected and measured using an optical read-out system (Supplemental material) (9, 10).

During the measurements, MTB is kept in traditional mycobacterial growth indicator tube (MGIT) media. A 21-h MTB AST protocol was developed recently for first-line antibiotics RIF and INH with a nanomotion device prototype (15). The technology has also been applied to other organisms (9–14, 16–20) and has been clinically validated for *Escherichia coli* and *Klebsiella pneumoniae* directly isolated from positive blood culture samples from bacteremia and sepsis patients (NANO-RAST, NCT05002413, manuscript in preparation (21), and Phenotech-1, NCT05613322 (10)). In this context, the nanomotion response to various antibiotic classes with different mechanisms of action (MoA), including cephalosporins, fluoroquinolones, macrolides, rifampicin, and others, have been described (10, 11, 13, 22).

This study examines the impact of the novel DprE1 inhibitory drugs PBTZ169, BTZ043, and their derivatives on MTB viability using the nanomotion technology platform in a BSL-3 setting. Although benzothiazinones interfere with mycobacterial cell wall integrity like the previously investigated bacteriostatic prodrug INH, they exhibit bactericidal properties and differ in target and MoA. Compared with previous studies with INH and RIF (15), we improved the time-to-result (TTR) from 21 h to 7 h, competitive with molecular diagnostics for MTB.

The activity of PBTZ169, H_2_-PPTZ169, BTZ043, and H_2_-BTZ043 (Material S1) were tested against the wild-type MTB strain H37Rv using a resazurin microtiter plate assay (REMA). Resazurin reduction to resorufin was used as a viability readout (23). MIC values were very low, with PBTZ169 showing the lowest (0.3 ng/mL) and H_2_-BTZ043 the highest (2.5 ng/mL) MIC, demonstrating high *in vitro* activity for all derivatives. The resistant-mutant NTB1 exhibited MICs > 50 µg/mL (Table 1).

**TABLE 1:** Compounds and their MICs according to the REMA assay

Compound	MIC for:	
	ATCC-25618/H37Rv	NTB1
PBTZ169 (MCZ)	0.3 ng/mL	>50 µg/mL
H_2_-PBTZ169	0.6 ng/mL	>50 µg/mL
BTZ043	1.0 ng/mL	>50 µg/mL
H_2_-BTZ043	2.5 ng/mL	>50 µg/mL

**Fig 2:**
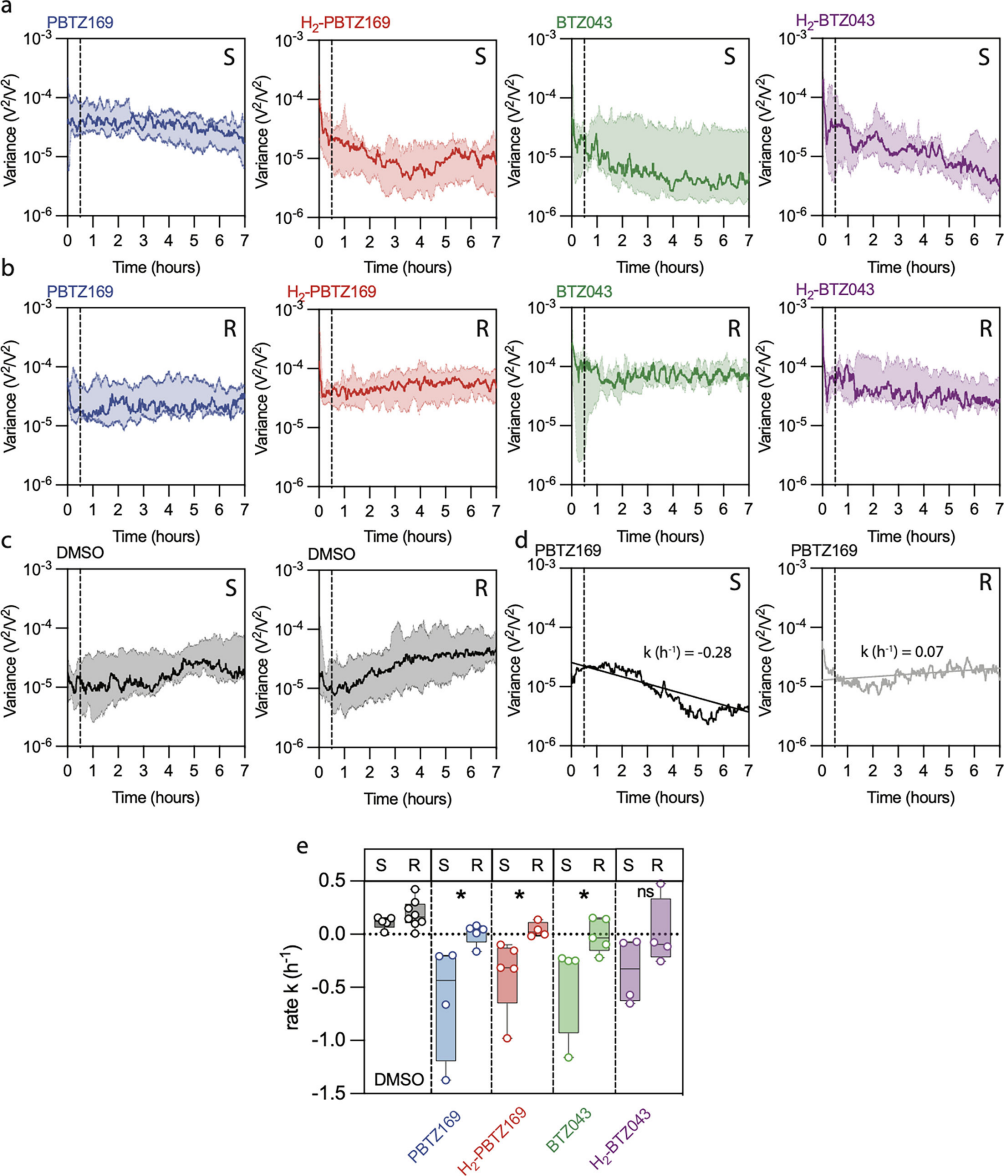
(a and b) Seven-hour nanomotion recordings include a 30-min medium phase (MGIT), followed by a 6.5-h drug phase with 0.2 µg/mL of PBTZ169 (blue), H₂-PBTZ169 (red), BTZ043 (green), and H₂-BTZ043 (purple) for the MTB reference strain ATCC-25618/H37Rv (S, susceptible) and mutant strain NTB1 (R, resistant). Median and interquartile ranges of cantilever deflection variance from ≥ 4 independent experiments are shown. The dashed line marks the start of the drug phase. Compounds were administered in 2 µL DMSO. (c) Median and interquartile range of variance over 7 h with only DMSO in the drug phase for both strains. (d) The variance of a representative replicate showing the rate k for both strains exposed to PBTZ169; k was calculated for each experiment separately over the entire 7 h recording. (e) Rates k of exponential fit of variance over 7 h for each experiment from (a-c), with boxes showing the median, minimum, and maximum values. Each data point represents the rate of one experiment. Groups were compared for significance using MWU. * indicates *P* = 0.0159.

We followed the recently established MTB nanomotion measurement protocol (15). The nanomotion prototype from that study was upgraded to the Phenotech device (10) with improved noise cancellation. Polydiallyldimethylammonium chloride (pDADMAC) was used as a linking agent to functionalize cantilevers to ensure stable MTB attachment to the cantilever throughout the experiment (Fig. S2), and nanoscale vibrations were measured over 7 h. After a 30-min recording in MGIT media (medium phase), 0.2 µg/mL of one of the four benzothiazinone derivatives was added, and the recordings continued for 6.5 h (drug phase). As a control, recordings were made for 6.5 h in a medium containing dimethyl sulfoxide (DMSO), as all drugs were prepared in a DMSO solution. The high concentration ensured a fast response to the antibiotic (5, 6) (Fig. 1). The experiment was further controlled by a blank phase, in which nanomotions of the bare cantilever were recorded before bacterial attachment. Here, we observed an order magnitude difference in the variance between the blank and medium phases (Fig. S3), indicating bacterial viability. In general, and as in previous nanomotion studies, the variance of the nanomotion signal over time was used as the primary readout (5, 6, 8, 9). For each molecule and H37Rv, we observed a decrease in variance over time, resulting in a negative slope (Fig. 2a). An exponential equation was fitted to obtain the rate constant k (h⁻¹) that was calculated according to the equation, log(*x*)  = log(*C*) + *kt*, where *t* is time, *k* is the rate of the common logarithm of the variance trend, and log(*C*) is the y-intercept. Within 7 h, each derivative exhibited a measurable impact on the variance, indicating an effect on the bacterial viability with median k between −0.43 h^−1^ (PBTZ169) and −0.25 h^−1^ (BTZ043). On the contrary, the DMSO control showed a clear increase in the variance over time with a median k = 0.12 h^−1^, which was significantly different from all samples with compound exposure (p_PBTZ169_ = p_BTZ043_ = p_H2-BTZ043_ = 0.0159, p_H2-PBTZ169_ = 0.0079, Mann-Whitney-U tests, MWU). The resistant *dprE1* mutant NTB1 exhibited median k between −0.03 h^−1^ (BTZ043) and 0.05 h^−1^ (PBTZ169), whereas DMSO resulted in a median k = 0.16 h^−1^. Despite sub-MIC concentrations, PBTZ169 and H_2_-PBTZ169 had a measurable impact on the viability when compared with DMSO due to higher reproducibility of the independent experiments (p_PBTZ169_ = 0.0186, p_H2-PBTZ169_ = 0.0485, MWU) contrary to BTZ043 and H_2_-BTZ043 with no significant impact (p_BTZ043_ = 0.0653, p_H2-BTZ043_ = 0.2141, MWU) (Fig. 2b through e).

Considering the development of an AST that repeatedly delineates susceptible and resistant bacterial responses to a given drug or compound, nanomotion recordings are combined with machine learning, which requires a far larger data set than currently available (10). However, the rate k is a valuable indicator for separating R and S phenotypes. PBTZ169, H_2_-PBTZ169, and BTZ043 exhibited significant differences in their k between R and S but not H_2_-BTZ043. PBTZ169 and H_2_-PBTZ169 achieved 100% separation between the S and R groups and, based on this metric, can be considered superior to BTZ043 and its derivative H_2_-BTZ043.

Several key factors are paramount in the battle against MDR and XDR MTB. Although MCZ offers hope in temporarily alleviating the bottleneck in TB therapy, its true potential can only be realized with rapid and precise diagnostics. Our results demonstrate a significant reduction in TTR for phenotypic AST, achieving TTRs as short as 7 h after culture positivity. This shortens the empirical treatment duration by up to 2 weeks compared with traditional phenotypic methods like broth microdilution, thus reducing the risk of incorrect antibiotic therapy and antimicrobial resistance selection pressure. Consequently, our approach brings phenotypic testing closer to molecular tests regarding speed (24, 25). The need for axenic MTB cultures for nanomotion experiments is a limitation; overcoming this could further reduce TTR by 2 weeks, the time typically required to reach culture positivity and commence AST. With nanomotion technology’s low inoculum requirement, AST directly from sputum samples is theoretically feasible, although the impact of commensal contamination must be assessed. If successful, this could enable AST from sputum in under a day. Our findings show that the nanomotion technology can be further enhanced by reducing environmental noise accomplished by the upgraded system compared with the prototype (TTR of 21 h for INH and RIF from culture) (15). This is further corroborated in a recent publication for *Enterobacteriaceae* reducing TTR from 4 to 2 h, incorporating temperature control and ML techniques (10). Expanding to a broader antibiotic panel and testing a variety of clinical isolates would enhance clinical utility, especially with regard to (novel) drug combinations standard in tuberculosis therapy (26); however, achieving this would require additional investment and further multiplexing of the Phenotech device, which extends beyond the scope of this study. With ongoing development, nanomotion technology could match molecular tests in TTR while uniquely providing viability information.

## SUPPLEMENTAL MATERIAL

Supplemental material aac.01318-24-s0001.docx Supplemental methods and Figures S1 to S3.

ASM does not own the copyrights to Supplemental Material that may be linked to, or accessed through, an article. The authors have granted ASM a non-exclusive, world-wide license to publish the Supplemental Material files. Please contact the corresponding author directly for reuse.
