# Supplementary material for: Real-time evaluation of macozinone activity against Mycobacterium tuberculosis through bacterial nanomotion analysis
Source: Antimicrob Agents Chemother. 2024 Nov 27;69(1):e01318-24. doi: 10.1128/aac.01318-24 (PMC11784433; doi:10.1128/aac.01318-24)
Supplement: Supplemental material — Supplemental methods and Figures S1 to S3. [file aac.01318-24-s0001.docx]

## **Supplementary Information**

## **Material and Methods**

### Bacterial strains, culture conditions, and antibiotic susceptibility tests

*Mycobacterium tuberculosis H37Rv* (ATCC® 25618), susceptible to macozinone (MCZ, including the derivatives PBTZ169, BTZ043, H_2_-PBTZ169, H_2_-BTZ043), and *M. tuberculosis* NTB1 carrying the Cys387Ser mutation in DprE1, resistant to MCZ (1), were used. Bacterial strains NTB1, a MCZ-resistant mycobacterial mutant, and H37Rv, MCZ-susceptible, were cultured in mycobacteria growth indicator tubes 7 mL (MGIT 960 Supplement Kit) and incubated using an automated mycobacterial detection system (BD BACTECTM MGITTM). Phenotypic susceptibility testing for both strains was conducted as previously described to determine susceptibility to MCZ [(2)]. Bacterial minimum inhibitory concentrations (MICs) were determined using resazurin microtiter plate assays (REMA).

### Resazurin Microtiter Plate Assays REMA

Mycobacterial strains were grown at 37°C in Mycobacteria Growth Indicator Tubes (MGIT, BD) supplemented with 0.2% glycerol, 0.05% Tween 80, and 10% oleic acid-albumin-dextrose-catalase (OADC). TB strains H37Rv and NTB1 were cultivated to mid-logarithmic phase in MGIT medium until an optical density at 600 nm (OD_600nm_) of approximately 0.5 was reached. The culture was subsequently diluted in MGIT medium to a theoretical OD_600nm_ of 0.0001. PBTZ169 and BTZ043 were resuspended in dimethyl sulfoxide (DMSO), and H_2_-PBTZ169 and H_2_-BTZ043 in degassed DMSO and subjected to two-fold serial dilutions. The drug solutions were then prepared for testing [(2)].

For the resazurin reduction microtiter assay (REMA), 100 μL of the diluted MTB H37Rv and NTB1 cultures were dispensed into each well of transparent flat-bottom 96-well plates. Serial dilutions of each drug were added to the wells. Each plate included control wells with media only without compound. The plates were incubated at 37°C for 6 days.

Following the incubation period, resazurin (0.025% [wt/vol]) was added to each well at a volume equal to 1/10 of the well volume. The plates were then incubated overnight. The fluorescence of the resorufin metabolite was measured using a TECAN Infinite M200 microplate reader with an excitation wavelength of 560 nm and an emission wavelength of 590 nm. The minimum inhibitory concentration (MIC_99_, referred to as MIC) was calculated using the Gompertz equation with GraphPad Prism software (version 10). Each drug was tested in at least triplicate to ensure reproducibility of results.

### Reagents and antibiotics

Dimethyl sulfoxide (DMSO), glycerol, Middlebrook 7H9, OADC, phosphate-buffered saline (PBS), polydiallyldimethylammonium chloride (pDADMAC), resazurin sodium salt and TWEEN 80, were purchased from Sigma Aldrich (St. Louis, MO). MGITs and MGIT 960 supplement kits were purchased from Becton Dickinson (Franklin Lakes, NJ). PBTZ169 and BTZ043 were dissolved in DMSO, while H2-PBTZ169 and H2-BTZ043 were dissolved in degassed DMSO. IM4TB provided the PBTZ169 and BTZ043, and their derivatives in powder form.

### Cantilever functionalization and bacterial attachment

To facilitate bacterial attachment and prevent cellular detachment during AST recording, we incubated the cantilever with 50 μl of  20% (v/v) pDADMAC for 5 min at room temperature (RT) (3). Following incubation, the pDADMAC drop was removed and discarded, after which the cantilever tip was gently washed with 100 μl of molecular biology-grade water. The sensors on the cantilever were then allowed to dry for at least 15 min before use.

MGIT cultured bacterial cells from prepared pellets needed to be immobilized onto the surface of the functionalized cantilever for nanomotion recording. First, pellets were resuspended in a PBS (Phosphate Buffer Saline, Corning) solution. Next, the sensor was placed on a clean layer of Parafilm® M (Amcor, Victoria, Australia). The tip of the sensor, containing the chip with the cantilever, was placed into contact with a single drop of bacterial cell suspension for 5 minutes. After this, the sensor was removed, gently washed with PBS, and assessed using phase microscopy for attachment quality (EVOS™ XL Core Imaging System). In the event of unsatisfactory attachment, the sensor was re-incubated in the cell suspension for an additional 30–60 s, or until satisfactory attachment was achieved. We aimed for an even bacterial distribution across the sensor. The attachment of bacteria is part of a filed patent (WO2021130339A1).

### Nanomotion measurement platform

The nanomotion measurement platform, the Resistell Phenotech device (Resistell AG, Muttenz, Switzerland), includes a stainless-steel head with a fluid chamber, an active vibration damping system, control electronics, and a computer terminal.

The platform uses atomic-force microscopy (AFM) technologies for antibiotic susceptibility testing (AST). Unlike standard AFM devices, the Phenotech device places the light source and photodetector below the cantilever to simplify the workflow. A light beam from a superluminescent diode (SLED) module (650 nm, 2 mW) is reflected off the cantilever and detected by a four-section photodetector, transforming flexural deflections into electrical signals. These signals are processed by custom electronics and recorded using a data acquisition card (USB-6212; National Instruments, USA). The device is managed with custom AST software (Resistell AG). The vertical movements of the cantilever (deflections) were recorded with a 60kHz acquisition rate.

Custom sensors (Resistell AG) with quartz-like, tipless cantilevers and gold coatings reflect the light beam. These cantilevers (SD-qp-CONT-TL, spring constant: 0.1 N/m, size: 130 × 40 × 0.75 µm, resonant frequency: 32 kHz in air; NanoWorld AG, Switzerland) deflect in response to bacterial movements during AST experiments.

### Nanomotion-recordings

Nanomotion-based AST was performed using Resistell Phenotech devices (Resistell AG, Muttenz, Switzerland) on a standard laboratory benchtop in a BSL-3 laboratory environment. Each recording has two phases: a 0.5-hour medium phase and a drug phase of 6.5 hours. Additionally, a short blank phase (5–10 minutes) measures baseline deflections of a new cantilever in PBS. The signal during the blank phase should be constant and flat (variance around 2.6 E-6 V^2^/V^2^ or lower). Higher values or peaks indicate possible contamination, sensor errors, or external noise, which should be corrected. Contamination (OD600 < 0.01) can cause deflection signals much higher than expected due to interactions between particles and the laser beam. The blank phase is a quality control step.

The medium phase records cantilever deflections after bacterial attachment, showing natural bacterial nanomotions from metabolic activity in MGIT medium. Variance here is higher (10^−5^ to 10^−3^ V^2^/V^2^) than in the blank phase. The 0.5-hour duration allows cells to adapt to the new environment and establishes a baseline for comparison with the following drug phase. The drug phase measures vibrations after introducing one of the macozinone derivatives into the measurement chamber. The antibiotic is directly added to the existing medium in the chamber.

### Calculating variance over time and slope analysis

Each nanomotion signal was divided into 10-second timeframes. Within each timeframe, the linear trend was removed, and the variance of the residual frame was estimated. In some experiments, the variance signal was too noisy for classification, necessitating an additional smoothing procedure. A running median with a 1-minute time window was applied to smooth the variance signal, facilitating an easier plot interpretation.

To calculate the rate of variance in the drug phase used to determine a drug's impact on bacterial nanomotions, we used the formula log(x) = log(C) + kt, where t represents time (in minutes), k is the rate of the common logarithm of the variance trend, and log(C) is the y-intercept.

### Biosafety

*M. tuberculosis* ATCC® 25618 and resistant MTB NTB1 strains were handled exclusively within the BSL-3 laboratory. The Resistell Phenotech AST instrument was positioned on a standard laboratory bench within the BSL-3 facility and connected to a computer via cable. All experiments were conducted by biologists who had received specialized BSL-3 training and were authorized to handle MTB strains.

## **Supplementary Figures**

**Supplementary Figure 1** Structural formulas of PBTZ169, H_2_-PBTZ169, BTZ043, and H_2_-BTZ043.

**
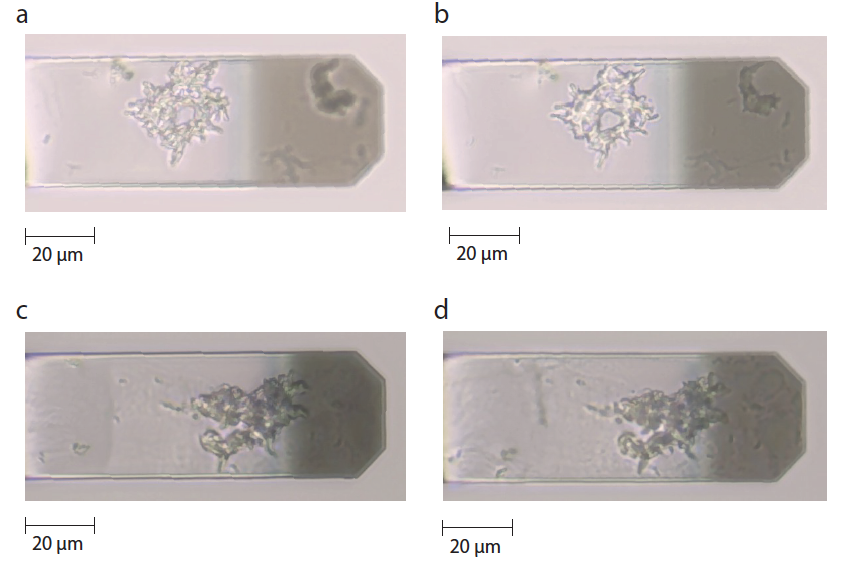
**

**Supplementary Figure 2. MTB attachment to cantilevers.** Cantilevers (NANOSENSORS™) measured 120–130 μm in length, 33–37 μm in width, and 720–780 nm in thickness. Prior to experimentation, cantilevers were incubated with MTB for 5 minutes. Attachment quality was assessed via phase contrast microscopy and then immediately placed in the measurement chamber to commence th nanomotion recording. Wild-type H37Rv (a) before and (b) after nanomotion recording. DprE1 mutant NTB1 (c) before and (d) after recording.

**
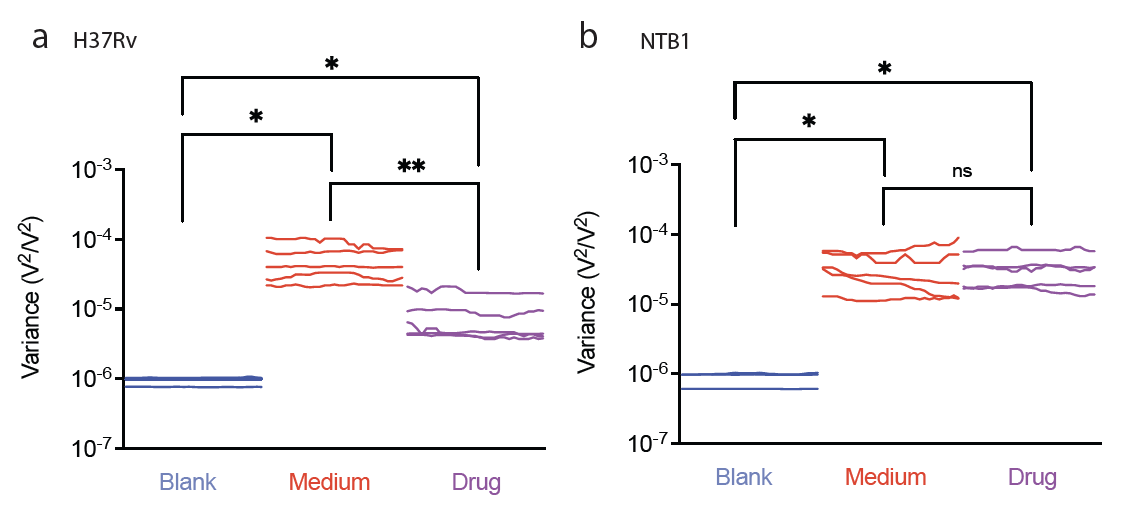
**

**Supplementary Figure 3**. Differences in the variance among the the last 5 minutes of the three different recording phases for the H37Rv susceptible strain: blank = empty cantilever, medium = bacteria attached to the cantilever in MGIT medium, drug = bacteria attached to the cantilever and exposed to 0.2 µg/ml PBTZ169. b) Same as a), but for the NTB1 resistant strain. The different phases were analyzed using Mann Whitney U test. ** indicates p = 0.0079, * indicates p = 0.0159, ns indicates p > 0.99.

**References**

1. Makarov, V., Manina, G., Mikusova, K., Mollmann, U., Ryabova, O., Saint-Joanis, B. *et al.* (2009) Benzothiazinones kill Mycobacterium tuberculosis by blocking arabinan synthesis Science **324**, 801-804 10.1126/science.1171583

2. Martin, A., Camacho, M., Portaels, F., andPalomino, J. C. (2003) Resazurin microtiter assay plate testing of Mycobacterium tuberculosis susceptibilities to second-line drugs: rapid, simple, and inexpensive method Antimicrob Agents Chemother **47**, 3616-3619 10.1128/AAC.47.11.3616-3619.2003

3. Smith, D. E., Dhinojwala, A., andMoore, F. B. G. (2019) Effect of Substrate and Bacterial Zeta Potential on Adhesion of Mycobacterium smegmatis Langmuir **35**, 7035-7042 10.1021/acs.langmuir.8b03920
